# Enhanced Conjugation of Auxin by GH3 Enzymes Leads to Poor Adventitious Rooting in Carnation Stem Cuttings

**DOI:** 10.3389/fpls.2018.00566

**Published:** 2018-04-26

**Authors:** Antonio Cano, Ana Belén Sánchez-García, Alfonso Albacete, Rebeca González-Bayón, María Salud Justamante, Sergio Ibáñez, Manuel Acosta, José Manuel Pérez-Pérez

**Affiliations:** ^1^Departamento de Biología Vegetal (Fisiología Vegetal), Universidad de Murcia, Murcia, Spain; ^2^Instituto de Bioingeniería, Universidad Miguel Hernández, Elche, Spain; ^3^Departamento de Nutrición Vegetal, Centro de Edafología y Biología Aplicada del Segura-Consejo Superior de Investigaciones Científicas, Murcia, Spain

**Keywords:** adventitious rooting, auxin homeostasis, auxin-conjugating enzymes, *Dianthus caryophyllus*, IAA degradation, polar auxin transport, stem cuttings

## Abstract

Commercial carnation (*Dianthus caryophyllus*) cultivars are vegetatively propagated from axillary stem cuttings through adventitious rooting; a process which is affected by complex interactions between nutrient and hormone levels and is strongly genotype-dependent. To deepen our understanding of the regulatory events controlling this process, we performed a comparative study of adventitious root (AR) formation in two carnation cultivars with contrasting rooting performance, “2101–02 MFR” and “2003 R 8”, as well as in the reference cultivar “Master”. We provided molecular evidence that localized auxin response in the stem cutting base was required for efficient adventitious rooting in this species, which was dynamically established by polar auxin transport from the leaves. In turn, the bad-rooting behavior of the “2003 R 8” cultivar was correlated with enhanced synthesis of indole-3-acetic acid conjugated to aspartic acid by GH3 proteins in the stem cutting base. Treatment of stem cuttings with a competitive inhibitor of GH3 enzyme activity significantly improved rooting of “2003 R 8”. Our results allowed us to propose a working model where endogenous auxin homeostasis regulated by GH3 proteins accounts for the cultivar dependency of AR formation in carnation stem cuttings.

## Introduction

New carnation (*Dianthus caryophyllus*) cultivars are mainly bred for traits affecting flower morphology, such as flower size, petal shape, petal number, flower color, and flower vase-life among others, as well as for traits improving plant production and pathogen resistance (Sheela, 2008). However, less attention is usually paid to breed the hidden-part of the plant, its root system, which is very important to warrant water and mineral nutrient supply. Commercial carnation cultivars are vegetatively propagated from axillary stem cuttings that undergo controlled rooting and acclimation (Garrido et al., 1996, 1998), which are high energy-demanding processes that lead to severe losses in certain cultivars (Agulló-Antón et al., 2011; Birlanga et al., 2015). Effective rooting of stem cuttings in several species strongly depends on the production of a functional adventitious root (AR) system, which is in turn affected by complex interactions between nutrient and hormone levels (Agulló-Antón et al., 2011; Díaz-Sala, 2014; Druege et al., 2016).

We previously performed a detailed analysis of the morphological and physiological changes occurring in the basal region of stem cuttings during rooting in a reference cultivar “Master”, and we reported how these were modified in response to exogenous auxin application (Agulló-Antón et al., 2014). We found significant crosstalk between auxin levels, stress hormone homeostasis, and sugar availability in the stem cutting base of “Master” during the initial steps of adventitious rooting (Agulló-Antón et al., 2014). To further characterize these interactions, we made use of a large collection of commercial carnation cultivars selecting two additional cultivars because of their contrasting rooting performance (Birlanga et al., 2015). The “2101–02 MFR” cultivar showed higher number of roots and faster growth than other spray cultivars, while the “2003 R 8” standard cultivar displayed a smaller root system because of a delay in root emergence and further slow root growth (Birlanga et al., 2015). We characterized gene expression and functional changes in the stem cutting base during the early stages of adventitious rooting in these two cultivars, which provided a number of molecular, histological, and physiological markers to initiate the genetic dissection of AR formation in this species (Villacorta-Martín et al., 2015).

In the current work, we performed a comparative study of AR formation in “2101–02 MFR”, “Master”, and “2003 R 8” grown *in vitro* and in soil plugs. We found that local auxin response in the stem cutting base was required for adventitious rooting, and that this local auxin maximum was dynamically established by active polar auxin transport (PAT) from the leaves. In turn, the bad-rooting behavior of the “2003 R 8” cultivar correlated with enhanced auxin inactivation in the root-formative region. Taking together, our results provide a detailed view of the major pathways triggering AR formation and how differential auxin homeostasis in the stem cutting base might account for cultivar-dependent adventitious rooting in carnation stem cuttings.

## Materials and Methods

### Plant Materials and Growth Conditions

Stem cuttings from the cultivars used in this work (“Master”, “2101–02 MFR”, and “2003 R 8”) are available upon request. All the mother plants had been grown in the same glasshouse under environmental conditions at 37°34’50^′′^ N, 1°46’35^′′^ W, and 395 m altitude within the rooting station of Barberet & Blanc, S.A. (Puerto Lumbreras, Murcia, Spain). Water, fertilizers, and adequate phytosanitary treatments were periodically applied, as described previously (Jawaharlal et al., 2009; Birlanga et al., 2015).

### Auxin Transport Inhibition

Terminal stem cuttings of about 10–15 cm with four to five pairs of leaves were manually harvested from several mother plants by skilled operators at the rooting station at noon on 26 April 2016, wrapped in plastic bags just after pinching and stored in a cold chamber at 5 ± 2°C for about 24 h, as described previously (Jawaharlal et al., 2009; Birlanga et al., 2015). A ring of lanolin paste (Sigma–Aldrich, United States) was individually set at about 6–8 mm of the basal end of each stem cutting using a syringe. Previously, warm lanolin was thoroughly mixed with 1% (w/w) of 1-naphthoxyacetic acid (1-NOA; Sigma–Aldrich, United States), 1% (w/w) of 1-*N*-naphthylphthalamic acid (NPA; Sigma–Aldrich, United States), or an equimolar mixture (0.8% w/w) of α-naphthalene acetic acid (NAA; Duchefa, Netherlands) and indole-3-butyric acid (IBA; Duchefa, Netherlands) for the different treatments. Non-supplemented lanolin paste was used as a mock treatment. After the treatments, the cuttings were individually planted in 104-well trays containing moistened peat/perlite (90/10 v/v) plugs (3.5 cm diameter × 3.5 cm length; ∼26 cm^3^) in a Gothic Arch Greenhouse at 38°16’43^′′^ N, 0°41’15^′′^ W, and 96 m altitude (Elche, Spain). Stem cuttings were grown from 27 April to 2 June 2016 under the environmental conditions of the greenhouse, with periodic sprinkler irrigation (5 min every 4 h).

For scoring adventitious rooting, the soil plug was carefully removed by washing it with high pressure tap water and the entire root system was imaged using a Nikon D3200 camera with an AF-S DX NIKKOR 18–55 mm f/3.5-5.6G VR objective. We visually defined seven rooting stages representing the different AR phenotypes observed (Birlanga et al., 2015).

### Auxin Transport Analysis

Three-to-five stem cutting basal sections (50 mm) from each carnation cultivar were used to estimate the basipetal indole-3-acetic acid (IAA) transport, as previously described (Garrido et al., 2002; Nicolás et al., 2007). The isolated stem sections were placed on top of an agar block keeping their endogenous apical–basal orientation and a 5 μl drop of a 200 μM labeled IAA ([^13^C]_6_C_4_H_9_NO_2_) was added to the top of each stem section. Every 30 min, the agar block was replaced by a new agar block and the experiment was carried out for 240 min. The amount of labeled IAA present in the agar blocks was analyzed by U-HPLC–MS Orbitrap (ThermoFisher Scientific, United States) using negative electrospray mode (ESI). Detection was made using the *m*/*z* ratio for labeled IAA (*m*/*z* 180.0761) and retention time to unequivocally identify transported labeled IAA. The linear traces of the cumulative labeled IAA transported per time unit were used to estimate different transport parameters according to Van der Weij (1932).

### Chemical Inhibition of Auxin Degradation

Terminal stem cuttings were collected from several mother plants at noon on 28 September 2017 and immediately placed on Erlenmeyer flasks filled with 50 mL of Murashige and Skoog salt media with Gamborg’s vitamins, pH 5.0 supplemented with 10 μM adenosine-5’-[2-(1H-indol-3-yl)ethyl]phosphate (AIEP) or with distilled water as a mock treatment. After 15 h in the dark, the cuttings were individually planted in 104-well trays and kept in a Gothic Arch Greenhouse as described above. Adventitious rooting stage and total root area were scored 29 days after planting as indicated elsewhere (Birlanga et al., 2015).

### Phytohormone Extraction and Analysis

Phytohormones were extracted and analyzed according to Großkinsky et al. (2014) and Villacorta-Martín et al. (2015). Auxin homeostasis metabolites were identified according to molecular mass and retention time from Total Ion Chromatograms obtained in the phytohormone analysis.

### RNA Isolation and First-Strand cDNA Synthesis

Sample collection and RNA extractions were performed as described elsewhere (Villacorta-Martín et al., 2015). Briefly, total RNA from ∼120 mg of powdered carnation stem tissue from 10 to 15 individuals was extracted in triplicate using Spectrum Plant Total RNA Kit (Sigma–Aldrich, United States) as previously described (Villacorta-Martín et al., 2015), and cDNA samples were synthesized from purified RNA using the iScript Reverse Transcription Supermix (Bio-Rad, United States). RNA extraction and cDNA synthesis were preformed according to the manufacturer’s instructions.

### Gene Expression Analysis by Real-Time Quantitative PCR

Primers were designed to amplify 87–178 bp of the cDNA sequences (**Supplementary Table S1**). To avoid amplifying genomic DNA, forward and reverse primers were designed to bind different exons and to hybridize across consecutive exons.

For real-time quantitative PCR, 14 μl reactions were prepared with 7 μl of the SsoAdvanced Universal SYBR Green Supermix (Bio-Rad, United States), 4 μM of specific primer pairs, and 1 μl of cDNA- and DNase-free water (up to 14 μl of total volume reaction). PCR amplifications were carried out in 96-well optical reaction plates on a Step One Plus Real-Time PCR System (Applied Biosystems, United States). Three biological and two technical replicates were performed for each gene. The thermal cycling program started with a step of 10 s at 95°C, followed by 40 cycles (15 s at 95°C and 60 s at 60°C), and the melt curve (from 60 to 95°C, with increments of 0.3°C every 5 s). Dissociation kinetics of the amplified products confirmed their specificity.

Primer pair validation was performed by using the 2^-ΔΔCT^ method (Livak and Schmittgen, 2001). Gene expression was measured by the absolute quantification method (Lu et al., 2012) by using a standard curve which comprised equal amounts from each cDNA sample. The *Dca3524* gene (homolog of the *Arabidopsis thaliana* housekeeping gene *EF1α*; AT5G60390) was chosen for normalization of the assayed genes, when needed. In each gene, mean of fold-change values relative to the distal region of the leaf in the “Master” cultivar (for the leaf data) and to the -23 h dataset in “2101–02 MFR” (for stem cutting base data) was used for graphic representation. Δ*C*_T_ values were analyzed using SPSS 21.0.0 (SPSS Inc., United States) by applying the Mann–Whitney *U*-test for statistical differences between cDNA samples (*P*-value ≤ 0.05).

### *In Silico* Identification of Candidate Genes From RNA-Sequencing Data and Heat Map Drawing

Sixty-nine differentially expressed genes (DEGs) identified from previous RNA sequencing (RNA-seq) data using the Short Time-Series Expression Miner (STEM) program (Villacorta-Martín et al., 2015) and annotated as GO:0009734 “auxin-activated signaling pathway” were initially selected; this list was further completed by including 35 genes from CarnationDB (Yagi et al., 2014) using keyword search (**Supplementary Table S2**). Sixty-six of these carnation genes were confirmed as putative auxin-responsive genes based on their highest homology with the *A. thaliana* annotation (Berardini et al., 2015). Following a similar approach, 49 genes were selected as cytokinin (CK)-related genes (GO:0009736 and GO:0009690) (**Supplementary Table S2**). Gene expression data from previous experiments (Villacorta-Martín et al., 2015) were processed using the heatmap.plus package of R^1^. Neighbor-joining distance matrixes between genes (rows) and between samples (columns) were calculated to build the dendrograms.

### Statistical Analyses

Statistical analyses were performed using the StatGraphics Centurion XV software (StatPoint Technologies, Inc., Warrenton, VA, United States). Data outliers were identified based on aberrant standard deviation values and excluded for posterior analyses. One-sample Kolmogorov–Smirnov tests were performed to analyze the goodness-of-fit between the distribution of the data and a given theoretical distribution as previously described (Chacón et al., 2013). Average ± standard deviation values were represented, except for those cases that did not exhibit a normal distribution and for which the median was used instead. The differences between the data groups were analyzed by *t*-test (*P* ≤ 0.05) when only two groups were compared. To compare the data for a given variable, we performed multiple testing analyses with a two-way ANOVA (cultivar × day after planting or cultivar × treatment) and the Tukey’s honestly significant difference (HSD) tests (*P* ≤ 0.05). Nonparametric tests were used when necessary.

## Results

### Adventitious Rooting in Carnation Stem Cuttings Is Genotype-Dependent

**FIGURE 1:**
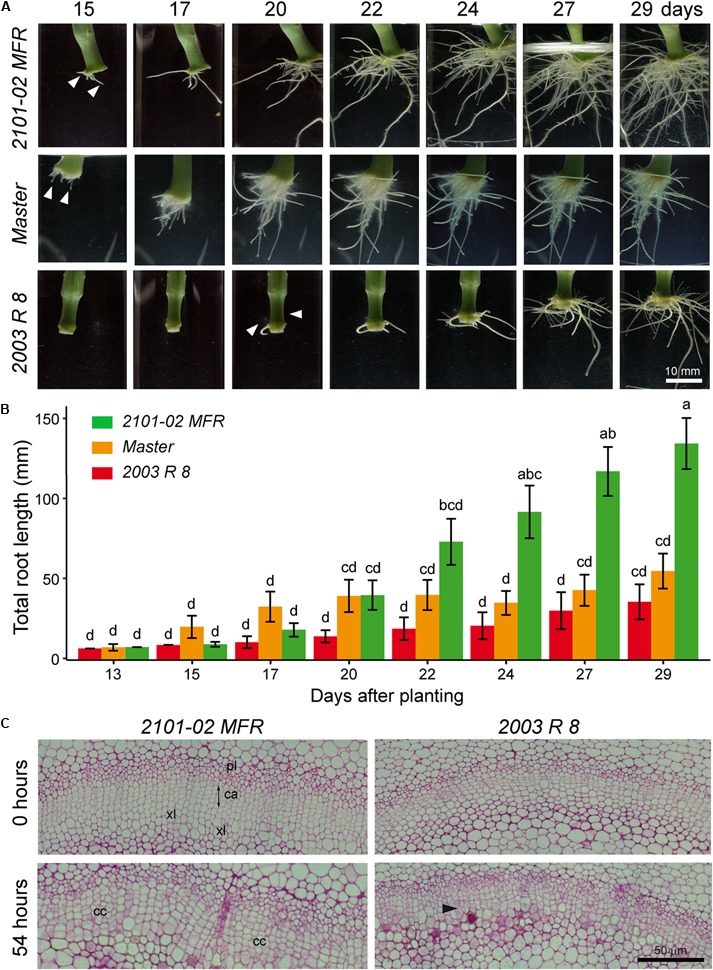
Cultivar-dependent adventitious rooting in carnation stem cuttings. **(A)** Time series of adventitious rooting in carnation stem cuttings grown *in vitro*. A representative sample in each cultivar was imaged between 15 and 29 days after planting. **(B)** The average lengths ± standard deviations of scanned root systems between 13 and 29 days are shown for the studied cultivars. Letters indicate significant differences (*P* < 0.05) between data points (*n* = 20). **(C)** Light micrographs from cross-sections of stem cutting basal regions at 0 and 54 h after planting. Black arrowhead indicates periclinal cell divisions in the cambium (ca). cc, cell clusters; pl: phloem; xl, xylem.

Using an environmentally controlled hydroponic system developed previously (Birlanga et al., 2015), we characterized the root system architecture during rooting of stem cuttings of two standard cultivars, “Master” and “2003 R 8”, and one spray cultivar, “2101–02 MFR” (**Figure 1**). In the “2101–02 MFR” and the “Master” cultivars, ARs emerged between 13 and 15 days after planting while there was a significant delay in AR emergence in the “2003 R 8” cultivar (**Figure 1A**). Although the three cultivars showed a well-developed and functional root system at 29 days, the “2101–02 MFR” cultivar showed a larger root system than the other two (**Figure 1A**). Considering total root length as an indicator of rooting performance (Birlanga et al., 2015), exponential root growth in “Master” initiated earlier (15 days) than in the two other cultivars: “2101–02 MFR” at 17 days and “2003 R 8” at 22 days (**Figure 1B**). The bad-rooting performance of “2003 R 8” was mostly caused by the severe growth delay of its root system compared to the other studied cultivars (**Figure 1B**). Conversely, the “2101–02 MFR” cultivar showed continuous root growth along the experiment with higher root growth rates (mm/day) than the two other cultivars (**Figure 1B**).

Periclinal cell divisions within the cambial ring were among the first morphological markers for the AR induction phase (de Klerk et al., 1999) observed in this species (Agulló-Antón et al., 2014). Discrete clusters of meristematic cells appeared along the cambial ring shortly afterward (Agulló-Antón et al., 2014), which correspond to AR initiation phase (de Klerk et al., 1999). Consistently with early AR initiation in “Master” and “2101–02 MFR”, cell clusters were observed within the vascular cambium at 54 h after planting (**Figure 1C**), while only sporadic periclinal divisions at the cambial ring were observed in “2003 R 8” growing in the hydroponic system (**Figure 1C**).

**FIGURE 2:**
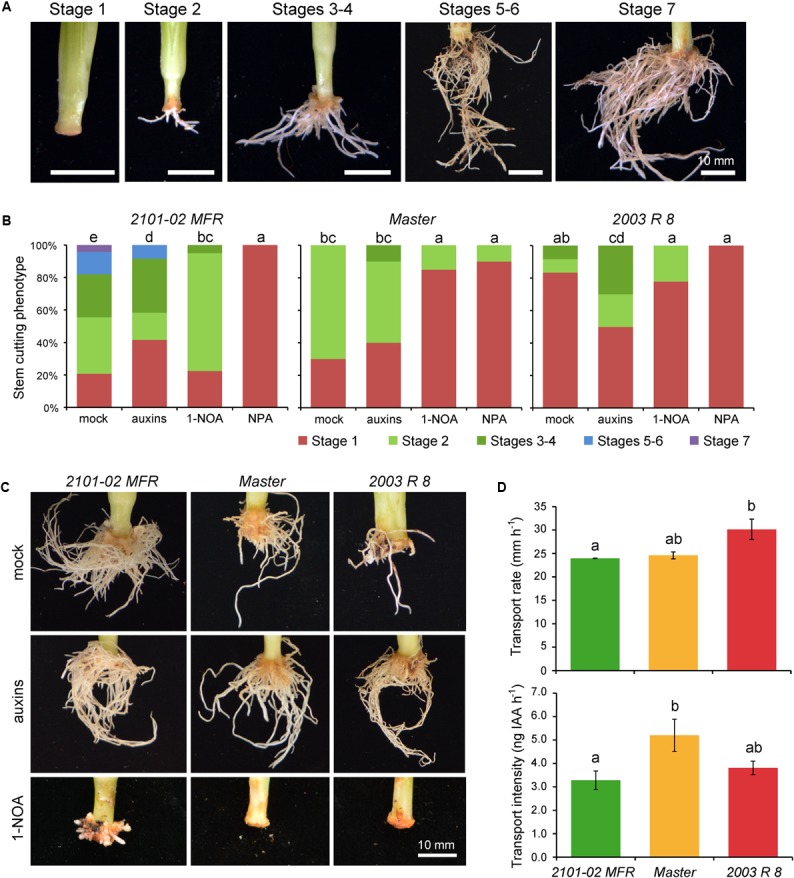
Auxin transport through the stem cutting base is required for adventitious root (AR) formation. **(A)** Representative images of adventitious rooting stages in carnation stem cuttings growing in soil plugs as defined previously (Birlanga et al., 2015). To visualize the entire root system, the soil substrate was carefully removed with pressurized water. **(B)** Graphic representations of rooting stage values in different carnation cultivars and treatments at 29 days, as described in Birlanga et al. (2015). Different letters indicate significant differences (*P* < 0.05) over sample means (cultivar × treatment). **(C)** Representative images of adventitious rooting in the studied carnation cultivars at 29 days treated with exogenous auxins or a polar auxin transport inhibitor (1-NOA). **(D)** Basipetal auxin transport parameters, transport rate (mm h^-1^), and transport intensity (ng IAA h^-1^) measured in stem cutting basal sections of the studied cultivars. Average ± standard deviation values are shown. Letters indicate significant differences (*P* < 0.05) over samples.

Based on a previously defined qualitative scale for rooting performance (**Figure 2A**; Birlanga et al., 2015), we confirmed the AR phenotypes of these three cultivars grown in soil plugs at the rooting station of our commercial provider. At 20 days, most stem cuttings in “Master” and “2101–02 MFR” were on stages 3 and 4, while only 60% of them reached stage 2 in “2003 R 8” (**Supplementary Figure S1A**). One week later, “Master” and “2003 R 8” cuttings reached similar rooting performances (**Supplementary Figure S1A**), but the root density in “2101–02 MFR” cuttings increased significantly (**Supplementary Figures S1A,B**). These results confirmed that the rooting behavior of “2003 R 8” (bad-rooting), “Master” (intermediate-rooting), and “2101–02 MFR” (good-rooting), despite it might be influenced by the environment and by the physiological status of the mother plants (Villanova et al., 2017), was under strict genetic control.

### Auxin Is Required for AR Initiation and AR Growth in Stem Cuttings

After stem cutting harvest, basipetal transport of auxin from mature leaves contributes to auxin accumulation in the stem cutting base and hence to AR formation (Garrido et al., 2002). In agreement with these results, exogenous auxin application improved AR formation in stem cuttings of “Master” (**Figures 2A–C**), likely by accelerating the formative divisions that lead to the establishment of the new root primordia (Agulló-Antón et al., 2014). The bad-rooting behavior of “2003 R 8” was rescued by exogenous auxin application, while a slightly negative effect of exogenously applied auxin was observed for adventitious rooting of “2101–02 MFR” cuttings (**Figures 2A–C**), leading to enhanced formation of callus-like tissue. However, the effect of the auxin treatment seems to be stronger at 29 days, because then the three cultivars showed similar root phenotypes (**Figure 2C**).

To confirm the relevance of a functional auxin transport through the stem for rooting, we chemically inhibited PAT either by NPA (Guerrero et al., 1999), which disrupts membrane trafficking (Geldner et al., 2001), or by 1-NOA, which blocked the activities of both auxin influx and efflux carriers (Parry et al., 2001; Lanková et al., 2010). Chemical disruption of PAT strongly inhibited AR formation in the stem cutting base in all three cultivars (**Figure 2B**). The inhibitory effect of NPA was stronger than that of 1-NOA, which also caused subtle cultivar-specific differences in AR development and growth, with incomplete inhibition of AR formation in “2101–02 MFR” (**Figure 2C**).

We next measured endogenous IAA transport through the stem using labeled IAA. The IAA transport rate was higher in “2003 R 8” than in the two other cultivars, while “Master” showed the highest IAA transport intensity (i.e., IAA mobilization) of the three (**Figure 2D**). In addition, we gathered the expression data of several genes putatively encoding auxin influx (*AUX/LAX*) and auxin efflux (*PIN* and *ABCB*) transporters (Oliveros-Valenzuela et al., 2008; Villacorta-Martín et al., 2015; Sánchez-García et al., 2018).

We validated these results by quantitative reverse transcription PCR (RT-qPCR) and extended them to additional time-points at -23 and -15 h. On the one hand, the expression of *DcAUX1* (*Dca32369*) and *DcLAX3* (*Dca6786*) auxin influx genes was not considerably changed over the rooting experiment in these two contrasting cultivars, although it was slightly higher in “2003 R 8” (**Supplementary Figures S2A,B**). On the other hand, the expression of auxin efflux genes, *DcABCB1* (*Dca43405*), *DcABCB19* (*Dca25164*), and *DcPIN1* (*Dca20927*), was higher in the stem cutting base at harvest time (-23 h) and diminished after planting in “2101–02 MFR” and specially in “2003 R 8” (**Supplementary Figures S2C–E**). In addition, the expression levels of *DcABCB1*, *DcABCB19*, and *DcPIN1*, were significantly higher in “2003 R 8” than in “2101–02 MFR” (**Supplementary Figures S2C–E**). These results indicated that the differences in PAT through the stem between “2101–02 MFR” and “2003 R 8” are likely caused by differential expression of auxin transporters, although these differences did not explain the contrasting rooting performance between “2003 R 8” and “2101–02 MFR”.

### Auxin Is Produced in Mature Leaves and Is Actively Transported to the Stem

**FIGURE 3:**
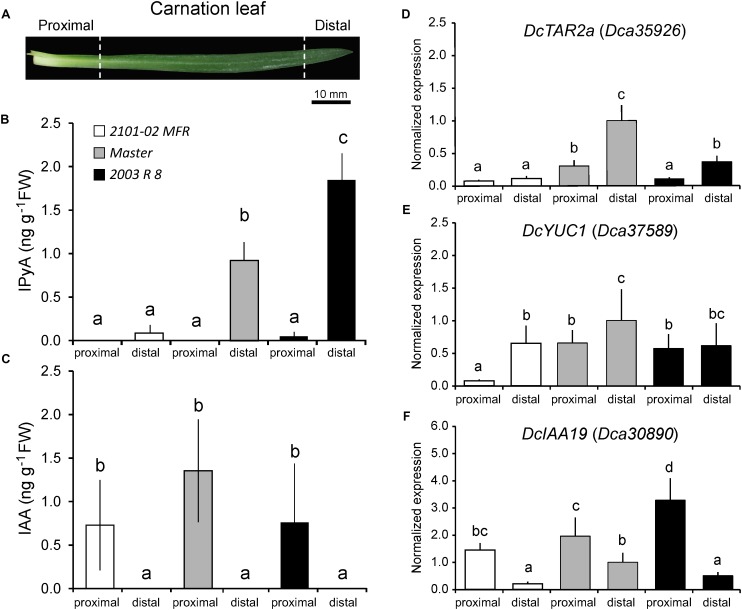
Differential auxin biosynthesis and auxin signaling in mature leaves at harvest. **(A)** Scheme of a carnation stem cutting leaf with indication of the two regions studied. **(B)** Indole-3-pyruvic acid (IPyA) and **(C)** indole-3-acetic acid (IAA) were measured in mature leaves of carnation stem cuttings at severance time. Average ± standard deviation values are shown. **(D,F)** RT-qPCR of the expression of selected transcripts related to **(D,E)** auxin biosynthesis or **(F)** auxin signaling in mature leaves of carnation stem cuttings at harvesting time. Bars indicate normalized expression levels ± standard deviation relative to the distal region of the leaf in the “Master” cultivar. Letters indicate significant differences between samples (*P* < 0.05).

The differences in AR formation between cultivars might arise by differential auxin production from mature leaves, as these are the main source of endogenous auxin required for rooting stem cuttings (Garrido et al., 2002). Carnation leaves are linear, display parallel venation, and are directly attached to the stem by a proximal sheath-like tissue, resembling monocot leaves (**Figure 3A**; Agulló-Antón et al., 2013). We measured endogenous auxin levels in proximal and distal regions of the leaf blade (**Figures 3A,B**). IAA was found at similar, albeit low levels, at the proximal region of the leaves in the three studied cultivars and it was below detection in their distal region (**Figure 3C**). In most plants, such as Arabidopsis and rice, the major IAA biosynthesis pathway occurs through indole-3-pyruvic acid (IPyA) (Zhao, 2010). The tryptophan aminotransferase (TAA/TAR) family produces IPyA and the YUCCA (YUC) family functions downstream in the conversion of IPyA to IAA (Mashiguchi et al., 2011; Won et al., 2011; Mano and Nemoto, 2012; Tivendale et al., 2014). Putative carnation orthologs *DcTAR2a* (*Dca35926*) and *DcYUC1* (*Dca37589*) were differentially expressed in proximal regions of mature leaves in all the three cultivars (**Figures 3D,E**). In addition, the expression of *DcTAR*2*a* and *DcYUC1* was significantly lower in “2101–02 MFR” than in “Master”, which was in agreement with the higher IAA levels found in the leaves of “Master” (**Figure 3C**). Consistent with local auxin biosynthesis occurring preferentially in the tip of the leaf, as it occurs in rice (Yamamoto et al., 2007), we confirmed that IPyA accumulated in the distal region of mature leaves, with significantly lower levels in “2101–02 MFR” leaves (**Figure 3B**). Auxin influx genes *DcAUX1* and *DcLAX3* were significantly expressed at higher levels in the proximal leaf region (**Supplementary Figures S3A,B**). The expression of genes involved in auxin efflux, such as *DcPIN1*, *DcABCB1*, and *DcABCB19*, was significantly higher in the proximal region of the leaves in the three cultivars (**Supplementary Figures S3C,E,F**). On the other hand, the carnation ortholog of *PIN3*, *Dca17139*, did not showed significant changes along the studied leaf regions (**Supplementary Figure S3D**). Similarly to that found for the stem (see above), combined expression levels of these auxin transporter genes were lower in “2101–02 MFR” than in the other two cultivars (**Supplementary Figure S3**), suggesting lower auxin transport rates also in the leaves of “2101–02 MFR”. To confirm the establishment of a gradient of IAA concentration within the leaf blade, we studied the expression of the auxin-induced *DcIAA19* gene (Villacorta-Martín et al., 2015) as a signaling read-out for the endogenous auxin gradient in the leaves (**Figure 3F**).

### Differential Accumulation of Active IAA in the Stem Cutting Base Between Cultivars Affects Rooting

**FIGURE 4:**
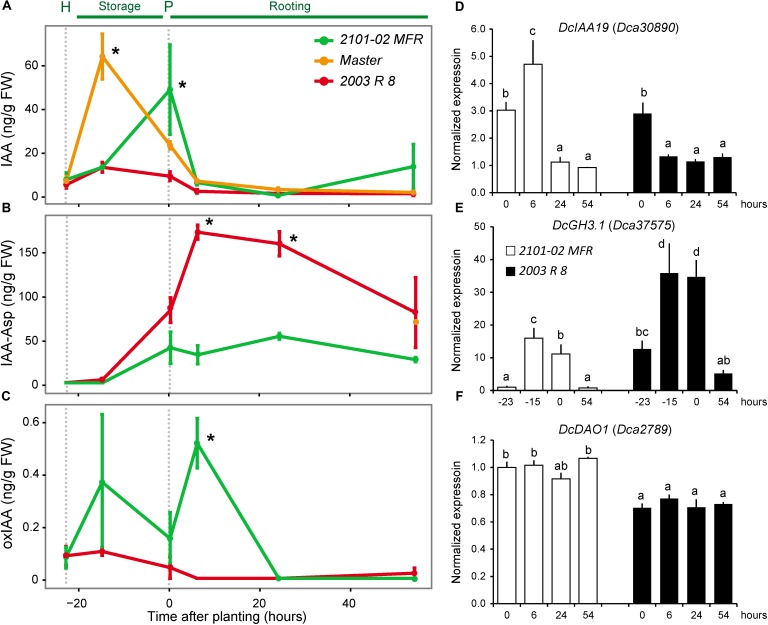
Endogenous levels of several auxin derivatives in the stem cutting base during adventitious rooting and expression of selected genes. **(A)** IAA, **(B)** indole-3-acetyl-L-aspartic acid (IAA-Asp), and **(C)** 2-oxo-indole-3-acetic acid (oxIAA). Average ± standard deviation values are shown at selected time-points: at harvesting time (H; –23 h), during storage at low temperature (–15 h), at planting time (P; 0 h), and during rooting (6, 24, and 54 h). Asterisks indicate significant differences between samples (*P* < 0.05). **(D–F)** Real-time PCR quantification of the expression of **(D)** *DcIAA19*, **(E)** *DcGH3.1*, and **(F)** *DcDAO1* relative to the –23 h dataset in the “2101–02 MFR” cultivar (not shown in the graph). Bars indicate normalized expression levels ± standard deviation. Letters indicate significant differences between samples (*P* < 0.05).

Auxin flooding in the vascular region above the wounding has been proposed to trigger de-differentiation and cell cycle reactivation of cambial cells in this region (Acosta et al., 2009). Endogenous IAA levels in the stem cutting base of “Master” transiently increased after harvesting and before planting from 8 to 65 ng g^-1^ FW (**Figure 4A**), similarly to as previously reported (Agulló-Antón et al., 2014). In “2101–02 MFR”, the IAA levels increased fivefold from steady-state levels up to 42 ng g^-1^ FW at planting time (**Figure 4A**). *DcYUC1* transcript levels were significantly downregulated in the stem cutting base of “2003 R 8” and “2101–02 MFR” after harvesting (**Supplementary Figure S2F**), and negligible IPyA concentrations were detected in these tissues. These results allowed us to discard the hypothesis that local auxin biosynthesis had contributed to the endogenous IAA accumulation found in the stem cutting base of these two cultivars after harvesting them from the mother plants.

Despite the highest PAT rate in the stem cutting base of “2003 R 8” (**Figure 2D**), its endogenous IAA level did not significantly increase after harvesting and during rooting (**Figure 4A**). These results suggested that the auxin maximum in the stem cutting base of “2003 R 8” was not properly formed. In *A. thaliana*, the main IAA degradation pathways (Ludwig-Müller, 2011) include oxidation by DIOXYGENASE FOR AUXIN OXIDATION 1 (DAO1) (Porco et al., 2016) and conjugation by GRETCHEN HAGEN 3 (GH3) amide synthetases (Staswick et al., 2005), which played highly redundant roles to regulate endogenous auxin levels (Mellor et al., 2016). To account for IAA degradation in the stem cutting base of the studied cultivars, we measured IAA conjugated to aspartic acid (IAA-Asp) and 2-oxoindole-3-acetic acid (oxIAA), which are metabolically inactive forms unable to be transported through the PAT system (Pencík et al., 2013). At harvest time (-23 h) and before rooting (-15 h), the levels of IAA-Asp in the stem cutting base of the three cultivars were low, but significantly increased at planting time and during rooting in “2003 R 8” (**Figure 4B**). On the other hand, oxIAA levels were low in the stem cutting base of these cultivars up to 54 h (**Figure 4C**). Remarkably, IAA-Asp levels in the stem cutting base of the bad-rooting cultivar, “2003 R 8”, increased threefold between planting time and 24 h (161.4 ± 14.1 ng g^-1^ FW) and returned to initial levels at 54 h (**Figure 4B**), which suggest further modification of the IAA-Asp pool by additional conjugations or oxidative reactions. OxIAA levels were not significantly different in the stem cutting base of “2003 R 8” during rooting, although some variation was found in oxIAA levels in “2101–02 MFR” during early rooting (**Figure 4C**).

We wondered whether the differences in endogenous IAA levels in the stem cutting base shortly after harvesting and before rooting might have a functional relevance for the rooting differences observed between cultivars. As described above, we used *DcIAA19* expression as a read-out for endogenous IAA levels. Interestingly, *DcIAA19* expression in the stem cutting base of the good-rooting cultivar (“2101–02 MFR”) matched its endogenous IAA levels (**Figure 4D**), while in the bad-rooting cultivar (“2003 R 8”), *DcIAA19* expression significantly decreased after planting (**Figure 4D**).

**FIGURE 5:**
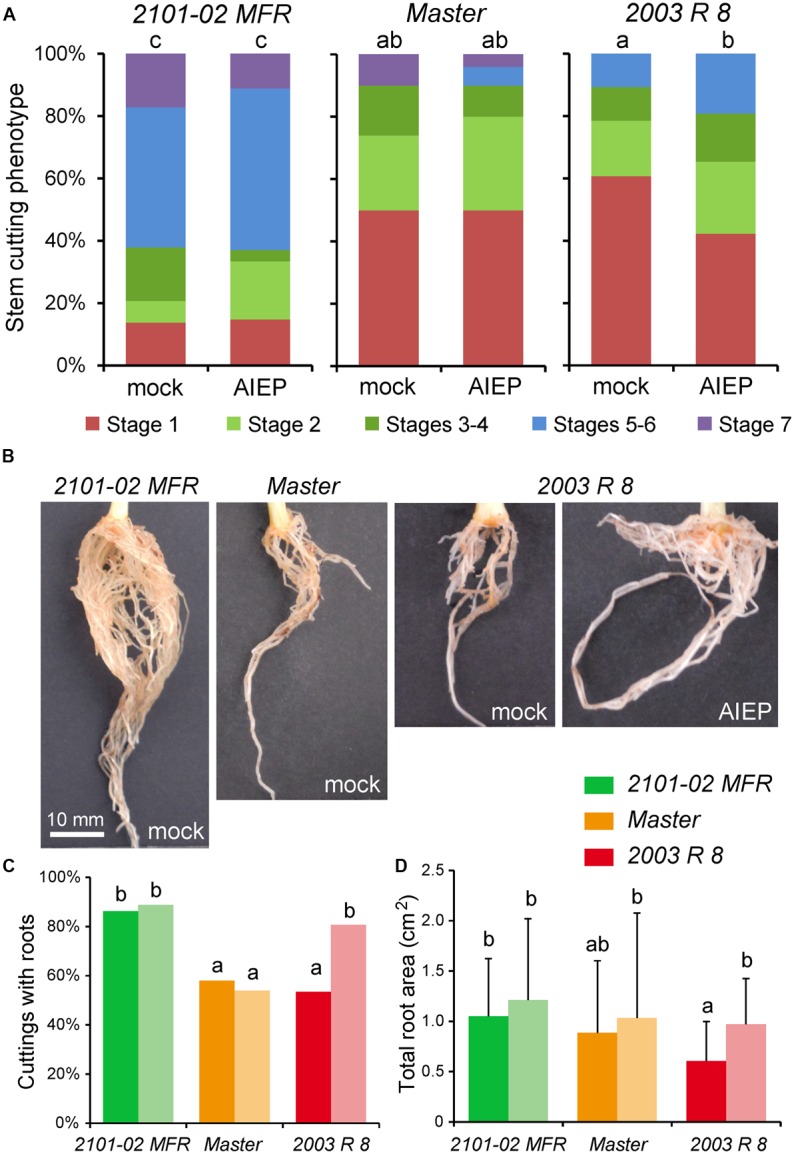
Auxin homeostasis at the stem cutting base is required for AR formation. **(A)** Graphic representations of rooting stage values in different carnation cultivars and treatments (*n* = 50). **(B)** Representative images of adventitious rooting in the studied carnation cultivars at 29 days treated with GH3 inhibitor (10 μM AIEP) or with mock. **(C,D)** The percentage of cuttings with roots **(C)** and the area of the scanned root system **(D)** at 29 days in rooted stem cuttings (*n* = 50). Dark- and light-colored bars represent data from mock- or AIEP-treated samples, respectively. Different letters indicate significant differences (*P* < 0.05) over sample means (cultivar × treatment).

We previously identified five carnation genes encoding GH3-like proteins whose orthologs synthesize IAA–amino acid conjugates, such as IAA-Asp (Staswick et al., 2005), two of which are known to be differentially expressed during rooting (Sánchez-García et al., 2018). The expression of *DcGH3.1* (*Dca37575*) was significantly higher in “2003 R 8” than in “2101–02 MFR”, with the strongest differences in expression after severance and before rooting (**Figure 4E**). In line with the endogenous oxIAA levels found in “2101–02 MFR” and “2003 R 8”, differences in gene expression levels were observed for the *DAO1* ortholog, *DcDAO1* (*Dca2789*) (**Figure 4F**). To confirm whether the differential accumulation of IAA-Asp in the studied cultivars contributed to rooting performance, we incubated freshly harvested stem cuttings of “2101–02 MFR”, “Master”, and “2003 R 8” with AIEP, a known chemical inhibitor of GH3 activities (Böttcher et al., 2012). Treatment with 10 μM AIEP did not significantly improve rooting performance either in the good-rooting cultivar “2101–02 MFR” or in “Master” (**Figure 5A**). In contrast, AIEP significantly improved the rooting performance of the bad-rooting cultivar “2003 R 8” (**Figure 5A**), which now produced more roots than in “Master” (**Figure 5B**). We found that the AIEP treatment increased both the percentage of cuttings with visible roots (**Figure 5C**) and the total root area (**Figure 5D**) in “2003 R 8”, with hardly any effect in the two other cultivars. Our results confirmed that GH3-mediated IAA-Asp accumulation in “2003 R 8” reduced IAA accumulation in the stem cutting base, which in turn negatively affects its rooting performance.

### Cytokinins and Stress-Related Hormone Levels in Cultivars With Contrasting Rooting Performance

Cytokinins are negative regulators of AR formation (de Klerk et al., 2001; Werner et al., 2003; Konieczny et al., 2009). We previously found that *trans*-zeatin (*t*Z) levels were high in the stem cutting base of “Master” after excision and they dropped abruptly after planting (Agulló-Antón et al., 2014). Consistently with these results, we found high *t*Z levels in the stem cutting base of “2101–02 MFR” and “2003 R 8” after excision (43.9 ± 15.8 and 34.8 ± 16.1 ng g^-1^ FW, respectively) that were quickly reduced in both cultivars before planting (**Supplementary Figure S4A**). From 24 h, *t*Z levels significantly increased in “2003 R 8” compared with those in “2101–02 MFR”, and remained at low levels during the experiment in this later cultivar (**Supplementary Figure S4A**).

Stem cutting excision from the mother plant alters the endogenous levels of the hormones regulating stress responses, particularly jasmonate (JA), abscisic acid (ABA), and ethylene (Agulló-Antón et al., 2014). In the stem cutting base of “Master”, endogenous levels of the metabolic precursor of ethylene, 1-aminocyclopropane-1-carboxylic acid (ACC), were low after excision and slightly increased during rooting (Agulló-Antón et al., 2014). While we found similar results regarding the ACC levels in the stem cutting base of “2101–02 MFR”, those in “2003 R 8” were constitutively and significantly lower during rooting (**Supplementary Figure S4B**). The increasing levels of ACC in “Master” and “2101–02 MFR” might reflect the decrease in endogenous ethylene production during adventitious rooting. The highest levels of endogenous ABA were found after cutting excision and during cold storage, with significant higher levels of ABA in “2101–02 MFR” than in “2003 R 8” (**Supplementary Figure S4C**). ABA levels diminished at planting time in both cultivars due to stem cutting rehydration and slightly increased during rooting (**Supplementary Figure S4C**).

### Auxin- and Cytokinin-Responsive Gene Expression Are Correlated With Rooting Performance

**FIGURE 6:**
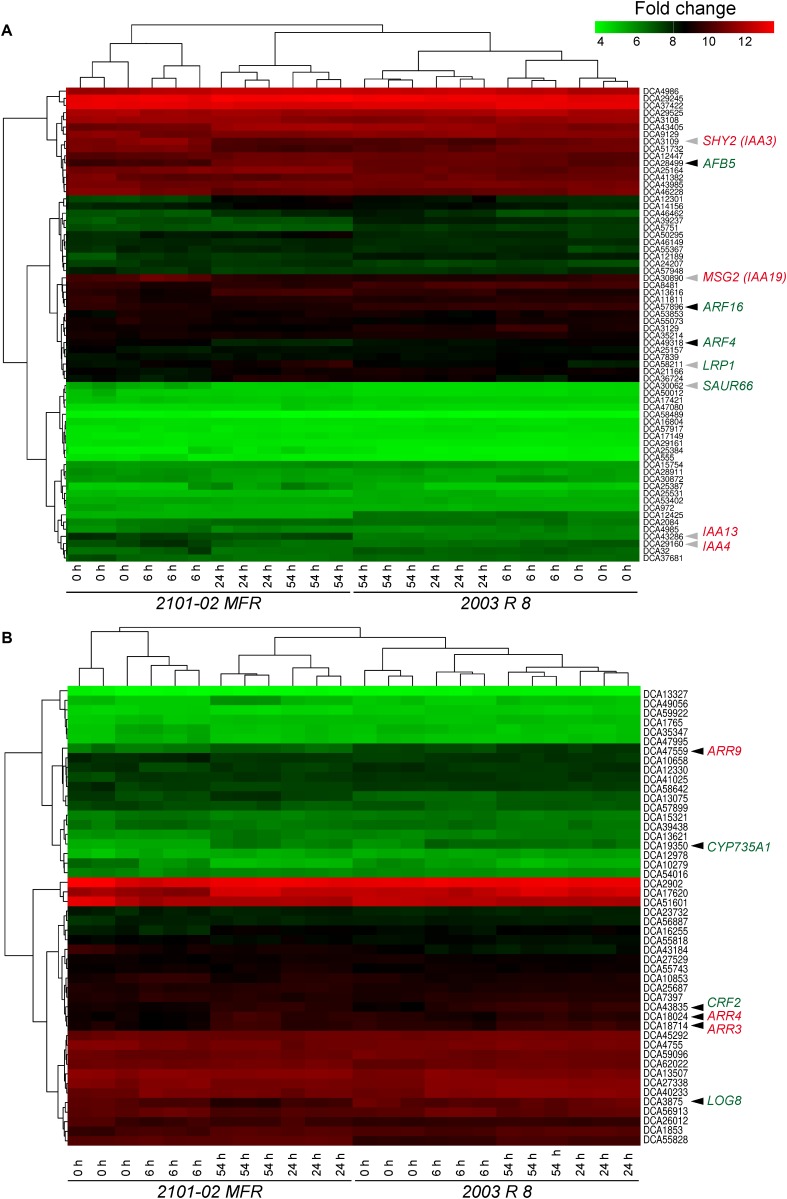
Analysis of expression of transcripts related to auxin **(A)** and cytokinin (CK) **(B)** homeostasis during AR formation. Heat map drawing and clustering was done as described in the section “Materials and Methods”. Arrowheads indicate those genes mentioned in the text with differential expression at defined time-points in *2101–02 MFR* (gray arrowheads) or “2003 R 8” (black arrowheads). Putative positive regulators are indicated in green, while negative regulators are shown in red.

Auxin signaling is mediated through the interaction of active auxin (e.g., IAA) with the TRANSPORT INHIBITOR RESPONSE 1/AUXIN SIGNALING F-BOX PROTEIN (TIR1/AFB) co-receptor and the Auxin/INDOLE-3-ACETIC ACID (Aux/IAA) transcriptional repressors, resulting in degradation of the Aux/IAAs and the release of AUXIN RESPONSE FACTOR (ARF) transcriptional partners (Lavy and Estelle, 2016). Some genes putatively encoding Aux/IAA repressors of auxin signaling, such as SUPPRESSOR OF HY2 MUTATION (SHY2), also named IAA3 (Tian et al., 2002) (*Dca3109*), IAA4 (*Dca29160*), or MASSUGU 2 (MSG2, also named IAA19; Tatematsu et al., 2004) (*Dca30890*), were expressed at higher levels in “2101–02 MFR” than in “2003 R 8” at 6 h (**Figure 6A**). In addition, other early auxin-responsive genes, such as *DcIAA13* (Weijers et al., 2005) (*Dca43286*), and *DcSAUR66* (*Dca30062*), were expressed at higher levels in “2101–02 MFR” across the experiment (**Supplementary Table S2**). Conversely, carnation genes putatively encoding *DcAFB5* (*Dca28499*), *DcARF4* (*Dca49318*), and *DcARF16* (*Dca57896*) were also expressed at higher levels in the stem cutting base of “2003 R 8” than in “2101–02 MFR” (**Supplementary Table S2**). Interestingly, the putative ortholog of the root-specific gene *LATERAL ROOT PRIMORDIUM 1* (*LRP1*; Smith and Fedoroff, 1995) (*Dca58211*) was expressed at higher levels in the stem cutting base of “2101–02 MFR” at 54 h, coinciding with cell cluster formation in this region (Villacorta-Martín et al., 2015). These results were consistent with the high amount of active IAA in the stem cutting base of “2101–02 MFR” at earlier time points during rooting (**Figure 4A**) that triggered AR formation.

Consistently with the high amount of *tZ* found in the stem cutting base of “2003 R 8” (**Supplementary Figure S4A**), the expression levels of *Dca19350*, the putative ortholog encoding CYP735A1 that catalyzes an early step of *tZ* biosynthesis in Arabidopsis (Takei et al., 2004), were increased in “2003 R 8” compared with those in “2101–02 MFR”, particularly during the first hours after planting (**Figure 6B**, and **Supplementary Figure S5A** and **Supplementary Table S2**). In addition, the expression of *Dca3875*, the closest carnation homolog to *LONELY GUY 8* (*LOG8*), which is also required for *t*Z biosynthesis (Kuroha et al., 2009), was also found at higher levels in the stem cutting base of “2003 R 8” (**Figure 6B** and **Supplementary Table S2**). Regarding the expression of other CK-related genes, carnation genes encoding some ARABIDOPSIS RESPONSE REGULATOR (ARR) proteins, such as *Dca18714*, *Dca18024*, and *Dca47559*, were expressed at high levels in the stem cutting base of “2003 R 8” shortly after planting, while in the good-rooting cultivar, “2101–02 MFR”, their expression levels were initially low and increased much later after planting (**Figure 6B**, and **Supplementary Figure S5B** and **Supplementary Table S2**). In Arabidopsis, the expression of *CK RESPONSE FACTOR* (*CRF*) genes is rapidly induced by CKs (Rashotte et al., 2006). We found that *Dca43835*, putatively encoding CRF2, was expressed at higher levels in the stem cutting base of “2003 R 8” than in “2101–02 MFR”, confirming higher CK signaling in the bad-rooting cultivar during early rooting (**Figure 6B**, and **Supplementary Figure S5C** and **Supplementary Table S2**).

## Discussion

We characterized the root system architecture of stem cuttings in two standard (“Master” and “2003 R 8”) and one spray (“2101–02 MFR”) carnation (*D. caryophyllus*) cultivars selected because of their contrasting rooting performance (Birlanga et al., 2015). We determined that the bad-rooting performance of “2003 R 8” was mostly caused by a severe delay in AR initiation compared to the other cultivars (Agulló-Antón et al., 2014; Villacorta-Martín et al., 2015). On the other hand, the highly developed root system of “2101–02 MFR” was caused by an early AR initiation and a sustained growth of its AR system.

**FIGURE 7:**
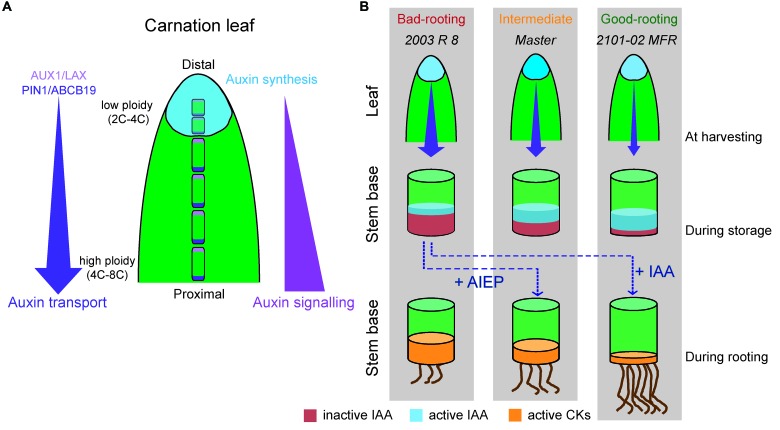
An integrated model of AR formation in carnation stem cuttings. **(A)** Regulation of auxin homeostasis in stem cutting leaves. Auxin influx proteins (AUX1/LAXs) are depicted in pink; auxin efflux proteins (PIN1 and ABCB19) are shown in purple. Size and ploidy level of mesophyll cells varied between distal (small size and 2C–4C ploidy level) and proximal (large size and 4C–8C ploidy level) leaf regions. The site of auxin synthesis is depicted in light blue. **(B)** Differences in auxin homeostasis in selected carnation genotypes during rooting of stem cuttings. In the leaf diagrams, the size of the purple arrow indicates the magnitude of the PAT from the leaves and the blue intensity indicates the level of auxin synthesis. In the stem cutting base diagrams, the height of the colored cylinders (red, blue, yellow) indicates the amount of hormone. IAA levels were highest during storage and were quickly downregulated after planting and during rooting. Treatment with AIEP (a well-known inhibitor of GH3 enzymes) results in a higher auxin-to-CK ratio at planting time that enhanced rooting in “2003 R 8” cultivar. Exogenous IAA treatment also enhanced rooting in the “2003 R 8” cultivar.

Auxin is mainly produced at the tip of the leaf through the IPyA pathway in most species (Cheng et al., 2007; Yamamoto et al., 2007; Liu et al., 2014) and it is then channeled through PAT to the vasculature and into the root (Gao et al., 2008; Naramoto, 2017). The standard cultivars “Master” and “2003 R 8” showed increased production of IPyA compared to the spray cultivar (“2101–02 MFR”), due to their higher expression of *DcTAR2a* in the most distal region of the leaf, with less differentiated cells (Agulló-Antón et al., 2013). IPyA is converted to IAA in the distal region of the leaf where *DcYUC1* is highly expressed, as this is the rate-limiting step of auxin biosynthesis (Zhao, 2012). We found very low IAA levels in the proximal region of the leaf in all the studied cultivars, suggestive of IAA mobilization via the PAT system preventing its accumulation in the leaf. In Arabidopsis, PIN1 is mainly expressed at the stele region of the root and the treatment with low concentrations of auxin up-regulates *PIN1* gene expression and modifies its protein localization (Vieten et al., 2005; Sauer et al., 2006; Omelyanchuk et al., 2016). These results suggest that endogenous auxin could directly affect the directionality and intensity of its own transport. Similarly, higher auxin biosynthesis in the standard cultivars (as measured by the amount of the IPyA precursor) could up-regulate PIN1 expression in the distal region of the leaves which, in turn, will increase PAT in these two cultivars. We found higher expression levels of several auxin influx genes (*DcAUX1* and *DcLAX3*) and auxin efflux genes (*DcPIN1*, *DcABCB1*, and *DcABCB19*) in the leaves of the cultivars with higher IPyA (and thus IAA) levels. These results suggested that “Master” and “2003 R 8” transported IAA through the leaf more efficiently than “2101–02 MFR”. Therefore, auxin was produced in the distal region of mature carnation leaves in all three cultivars at a different pace where it was quickly mobilized by PAT toward their proximal region (**Figure 7**). As a consequence, a gradient of IAA concentration along the leaf blade could be established, which was visualized by *DcIAA19* expression, used previously as a read-out of endogenous auxin levels (Villacorta-Martín et al., 2015).

We found that IAA transport through the stem was higher in “Master” and “2003 R 8” than in “2101–02 MFR”, which mirrored the expression of their auxin transport genes in this tissue. Auxin transport inhibitors block PIN cycling between the plasma membrane and endosomal compartments (Geldner et al., 2001; Parry et al., 2001). Inhibition of PAT through the stem with NPA abolished AR formation in all three cultivars, while the auxin influx inhibitor 1-NOA strongly affected “Master” and “2003 R 8”. These cultivar-specific responses to PAT inhibitors might be caused by the differential expression of auxin transport genes, which were much lower in the “2101–02 MFR”. Indeed, the reduced rate of auxin biosynthesis in the leaves and the slower PAT in the stem cutting base of “2101–02 MFR” might contribute to the temporal delay and its lower IAA concentration found after harvesting compared to that of “Master”. However, the contrasting rooting behavior of “Master” and “2003 R 8” could not be explained because of their differences in auxin biosynthesis in mature leaves or in their auxin transport rates through the stem. Exogenously added auxins enhanced AR formation in “Master” and they partially restored the bad-rooting behavior of “2003 R 8”, indicating that both cultivars are similarly responsive to auxin.

Interestingly, despite the high auxin biosynthesis and transport rate in “2003 R 8”, the localized auxin accumulation in the stem cutting base required for rooting was not observed, suggesting altered auxin homeostasis (i.e., auxin degradation) in the stem cutting base of “2003 R 8”. The dynamic regulation of IAA oxidation by the DAO1 family of dioxygenases and of amino acid IAA conjugation by GH3 represents the major contribution to auxin homeostasis in Arabidopsis (Mellor et al., 2016; Porco et al., 2016). We found very low oxIAA levels in the stem cutting base of the studied cultivars which otherwise was not detected in leaves (Sánchez-García et al., 2018), indicating that IAA oxidation is not involved in the main regulatory pathway of IAA homeostasis in carnation. The expression of *DcDAO1* genes was constitutively low and remained unchanged during rooting. As oxIAA can be further metabolized by conjugation to glucose, one possibility is that glucosylation of oxIAA to 2-oxoindole-3-acetic acid glucose (oxIAA-Glc) by the UDP glucosyltransferase UGT74D1 (Tanaka et al., 2014) reduced endogenous oxIAA levels. However, we could not detect oxIAA-Glc in the stem cutting base of these cultivars, despite of the high expression of DcUGT74D1 in this tissue (Sánchez-García et al., 2018), which confirmed the low relevance of the oxIAA pathway to explain varietal differences during rooting of carnation stem cuttings.

Several forms of auxin conjugates have been identified in plants, including IAA-sugar (i.e., IAA-Glucose and IAA-Glc) and IAA–amino acid conjugates (Ludwig-Müller, 2011). The conversion of IAA to IAA-Glc is catalyzed by the UDP glucosyltransferase UGT84B1, and its overexpression in Arabidopsis caused phenotypes compatible with auxin depletion, suggesting that IAA-Glc is an irreversible IAA catabolite rather than as an intermediate in the synthesis of other conjugates (Jackson et al., 2002). IAA-Glc was not detected either in leaves or in the stem cutting base of the studied cultivars, indicating that IAA-Glc conjugates might represent relatively minor components of the conjugate pool in carnation (Sánchez-García et al., 2018). The conjugation of IAA to amino acids, such as aspartic acid and glutamic acid, is catalyzed by a group of IAA–amido synthetases belonging to the family of GH3 proteins (Staswick et al., 2005). We found low levels of IAA-Asp both in the leaves and in basal region of the stem in cuttings harvested from the mother plants (Sánchez-García et al., 2018). Interestingly, IAA-Asp levels in the stem cutting base of “2003 R 8” progressively increased after planting and during rooting. On the other hand, endogenous IAA-Asp levels remained low and constant during rooting in “2101–02 MFR”. Although the expression of *DcGH3.1* was strongly up-regulated in the stem cutting base of both cultivars during rooting, the highest expression in the stem cutting base of “2003 R 8” might increase its endogenous GH3 activity leading to the observed IAA reduction after harvesting and before planting.

Indole-3-acetic acid conjugated to aspartic acid is believed to be inactive (Ludwig-Müller, 2011); hence, high IAA-Asp might interfere with the build-up of the localized auxin response required for AR formation in the stem cutting base of “2003 R 8”. Previous results in pea suggested that there are high levels of IAA inactivation in the base of mature cuttings compared with the juvenile ones, which might directly contribute for the observed decline in AR formation with age in this species (Rasmussen et al., 2015). To confirm our hypothesis that enhanced IAA-Asp conjugation in the stem cutting base reduced rooting of “2003 R 8”, we applied AIEP, a known inhibitor of the GH3 family of enzymes that it has been shown to be effective *in vivo* (Böttcher et al., 2012). Treatment of stem cuttings with AIEP significantly improved rooting of “2003 R 8”. The percentage of AIEP-treated cuttings with roots increased significantly only in “2003 R 8” compared to the non-treated ones and without any effect on the other studied cultivars, indicating that the active IAA levels in the stem cutting base required for AR initiation were only limiting in the bad-rooting cultivar (“2003 R 8”). Hence, the delay in AR initiation of “2003 R 8” was fully rescued by chemically inhibiting GH3 activities, confirming that enhanced auxin homeostasis in the stem cutting base of “2003 R 8” was responsible for its bad-rooting performance. It might be possible that other bad-rooting cultivars could also accumulate IAA-Asp in the stem cutting base during storage and that AIEP treatment might improve adventitious rooting. Preliminary results indicate that other standard cultivars with poor rooting performance, such as “2441-7 R” (Birlanga et al., 2015), also responded to the AIEP treatment (M.S. Justamante and J.M. Pérez-Pérez, personal communication).

Several Aux/IAA genes were expressed at high levels in the stem cutting base of “2101–02 MFR” during early rooting. Due to most Aux/IAA genes are auxin-inducible (Overvoorde et al., 2005; Paponov et al., 2008), the Aux/IAA function is regulated by endogenous auxin at both protein stability and gene expression level (Pierre-Jerome et al., 2013). In Arabidopsis, several Aux/IAA gain-of-function mutants, such as *msg2/iaa19* (Tatematsu et al., 2004), *shy2/iaa3* (Tian et al., 2002), *bodenlos/iaa12* (Hamann et al., 2002), or *solitary root/indole-3-acetic acid14* (Fukaki et al., 2002), are auxin insensitive and are defective in LR formation, indicating that multiple Aux/IAA–ARF modules cooperatively regulate the developmental steps during LR formation (De Smet et al., 2010; Goh et al., 2012). Some of these Aux/IAA–ARF modules are also conserved during AR formation from whole leaves (Bustillo-Avendaño et al., 2017). Hence, the high expression levels of *DcIAA3*, *DcIAA13* (the closest ortholog of *BDL/IAA12*), and *DcIAA19* in the stem cutting base of the good-rooting cultivar might indicate low levels of these three repressors after PAT-induced auxin accumulation in the formative region of the stem. Conversely, lower IAA levels in the stem cutting base of the bad-rooting cultivar (“2003 R 8”) might allow the DcIAA co-repressors to accumulate and hence inhibit rooting. Intriguingly, we found *DcAFB5* and some *DcARFs* were expressed at higher levels in the stem cutting base of “2003 R 8”, which might indicate a compensatory mechanism to regulate high DcIAA activity. It is tempting to speculate that ARF dimerization in the stem cutting base of “2101–02 MFR” will thus activate the expression of specific targets involved in AR initiation more efficiently than in “2003 R 8”, one of which might be *LRP1*, which is 1 of the 10 members of the *SHI* gene family (Kuusk et al., 2006). *LRP1* is induced by auxin and expressed in cells derived from the pericycle and appears to be active both in LR and AR formation (Welander et al., 2014).

We found that CK levels (*t*Z) in the stem cutting base of the bad-rooting cultivar (“2003 R 8”) increased during rooting at higher levels than in the other studied cultivars, which was correlated with the higher expression of two *t*Z biosynthesis genes (Kiba et al., 2013), *DcCYP735A1* and *DcLOG8*. Notably, a negative role for CK in AR formation has been proposed as mutants defective in CK biosynthesis or perception displayed increased production of ARs whereas enhanced CK biosynthesis has the opposite effects (de Klerk et al., 2001; Werner et al., 2003; Riefler et al., 2006; Ramírez-Carvajal et al., 2009). In agreement with a negative role of CKs in AR formation, two ARR CK response genes, *DcARR3* and *DcARR9*, were significantly upregulated in the stem cutting base of “2003 R 8”. ARR3 and ARR4 are type-A of response regulators (Hwang et al., 2012) that are quickly induced by CKs and that redundantly regulate branching patters in the shoot in response to nitrate availability (Müller et al., 2015). Recently, ammonium has been shown to improve adventitious rooting in leafy cuttings of *Petunia hybrida* (Hilo et al., 2017). Further investigations will clarify the role of *DcARR3* and *DcARR4* in nutrient-induced AR formation in carnation stem cuttings.

Our deep understanding of the physiological and molecular events leading to the specific developmental responses of AR formation in “2101–02 MFR” and “2003 R 8” cultivars will allow establishing a marker-assisted selection approach of DEGs to select for enhanced adventitious rooting traits during breeding and to limit production losses during vegetative propagation of elite lines.

## Author Contributions

MA and JMP-P were involved in the conceptualization and supervision. AC, AA, MA, and JMP-P performed the methodology. AC, ABS-G, AA, RG-B, MSJ, and SI were involved in the investigation. AC, MA, and JMP-P performed the formal analysis. JMP-P was involved in the writing of the original draft. AC, MA, and JMP-P were involved in the writing and review and editing of the manuscript. JMP-P provided the funding acquisition. AA, MA, and JMP-P collected resources for the study.

## Conflict of Interest Statement

The authors declare that the research was conducted in the absence of any commercial or financial relationships that could be construed as a potential conflict of interest.

## Footnotes

1 http://www.r-project.org/

## Supplementary Material

FIGURE S1 Rooting stages in carnation stem cuttings grown in soil plugs. **(A)** Stacked histograms of rooting stages in a representative sample (*n* = 50) of stem cuttings growing in soil plugs for 20 or 27 days after planting. Letters indicate significant differences (*P* < 0.05) over samples (cultivar × time). **(B)** Representative soil plug images of stem cuttings rooting for 20 or 27 days.

FIGURE S2 Real-time PCR quantification of the expression of selected transcripts related to auxin transport (auxin influx, **A,B**; auxin efflux, **C,E**) or auxin biosynthesis **(F)** in the stem cutting base during adventitious rooting. Bars indicate normalized expression levels ± standard deviation relative to the -23 h dataset in the “2101–02 MFR” cultivar. Letters indicate significant differences between samples (*P* < 0.05).

FIGURE S3 Real-time PCR quantification of the expression of selected transcripts related to auxin transport (auxin influx, **A–B**; auxin efflux, **C–F**) in mature leaves of carnation stem cuttings at harvesting time. Bars indicate normalized expression levels ± standard deviation relative to the distal region of the leaf in the ‘Master’ cultivar. Letters indicate significant differences between samples (*P* < 0.05).

FIGURE S4 Endogenous levels of other key hormones in the stem cutting base during adventitious rooting. **(A)** *trans*-zeatin (*t*Z), **(B)** the ethylene precursor 1-aminocyclopropane-1-carboxylic acid (ACC), and **(C)** abscisic acid (ABA). Average ± standard deviation values are shown. Asterisks indicate significant differences (*P* < 0.05) over time for a given treatment. H, harvesting; P, planting.

FIGURE S5 Real-time PCR quantification of the expression of selected transcripts related to CK biosynthesis **(A)** or signaling **(B,C)** in the stem cutting base during adventitious rooting. Bars indicate normalized expression levels ± standard deviation relative to the -23 h dataset in the “2101–02 MFR” cultivar. Letters indicate significant differences between samples (*P* < 0.05).

TABLE S6 Oligonucleotides used in this study.

TABLE S7 RNA-seq data from carnation genes selected as putative auxin-responsive and CK-related genes.
