# Supplementary figures and images for: Enhanced Conjugation of Auxin by GH3 Enzymes Leads to Poor Adventitious Rooting in Carnation Stem Cuttings
Source: Front Plant Sci. 2018 Apr 26;9:566. doi: 10.3389/fpls.2018.00566 (PMC5932754; doi:10.3389/fpls.2018.00566)

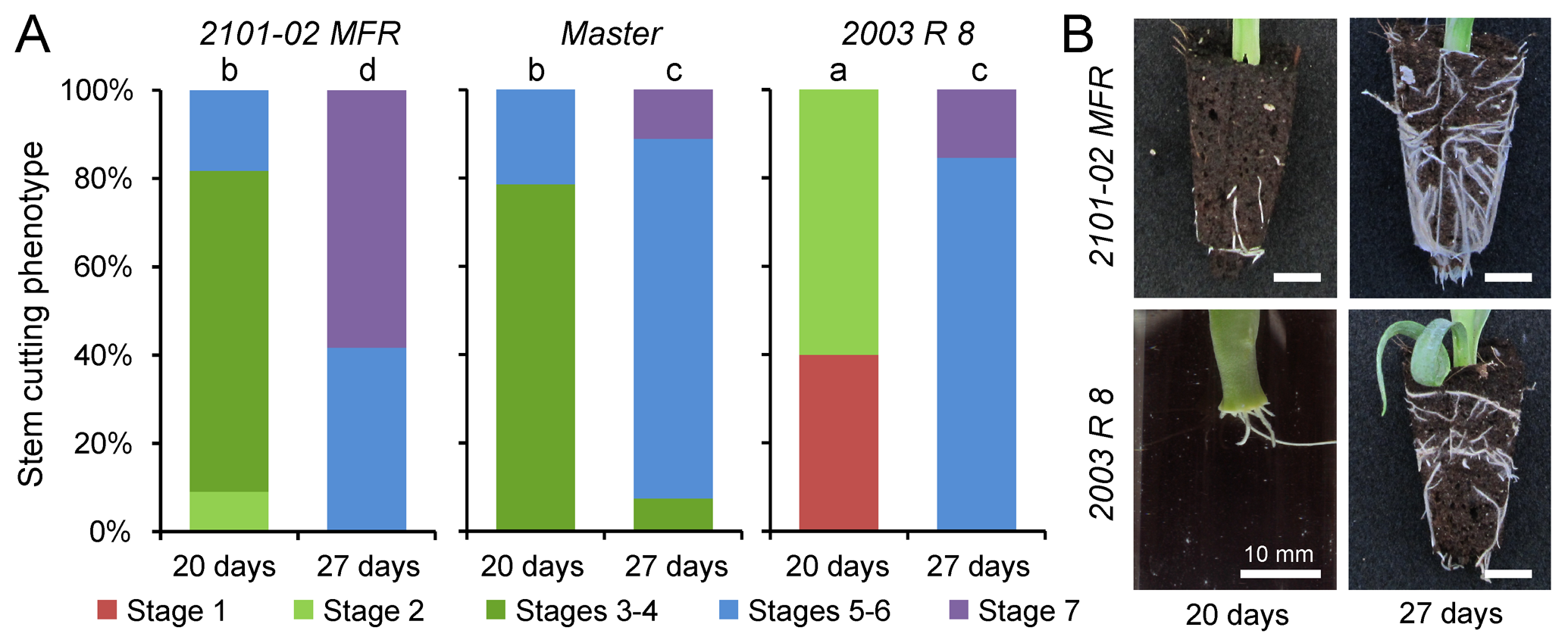

Supplement: FIGURE S1 — Rooting stages in carnation stem cuttings grown in soil plugs. (A) Stacked histograms of rooting stages in a representative sample (n = 50) of stem cuttings growing in soil plugs for 20 or 27 days after planting. Letters indicate significant differences (P < 0.05) over samples (cultivar × time). (B) Representative soil plug images of stem cuttings rooting for 20 or 27 days. [file Image_1.TIF]

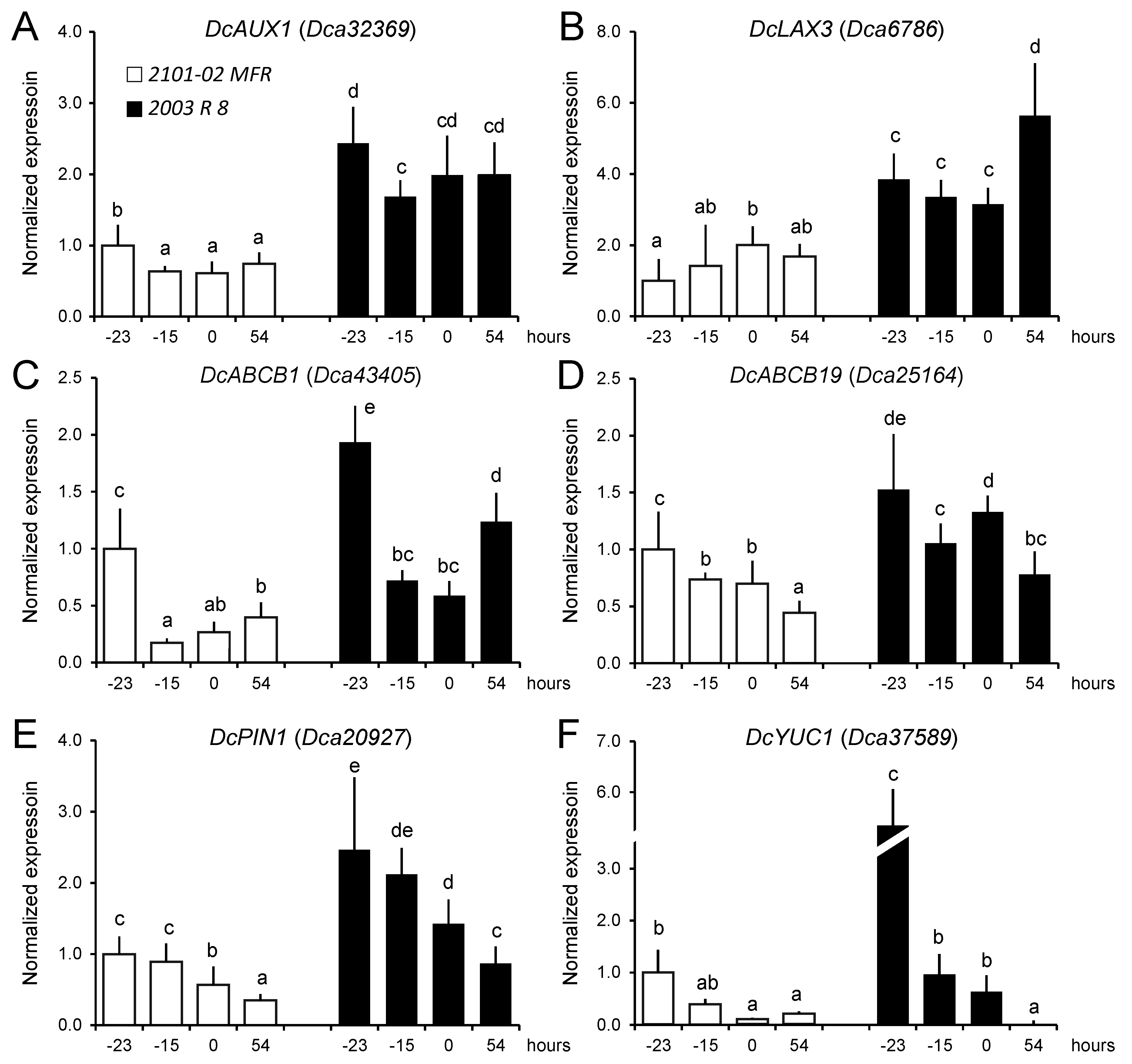

Supplement: FIGURE S2 — Real-time PCR quantification of the expression of selected transcripts related to auxin transport (auxin influx, A,B; auxin efflux, C,E) or auxin biosynthesis (F) in the stem cutting base during adventitious rooting. Bars indicate normalized expression levels ± standard deviation relative to the -23 h dataset in the “2101–02 MFR” cultivar. Letters indicate significant differences between samples (P < 0.05). [file Image_2.TIF]

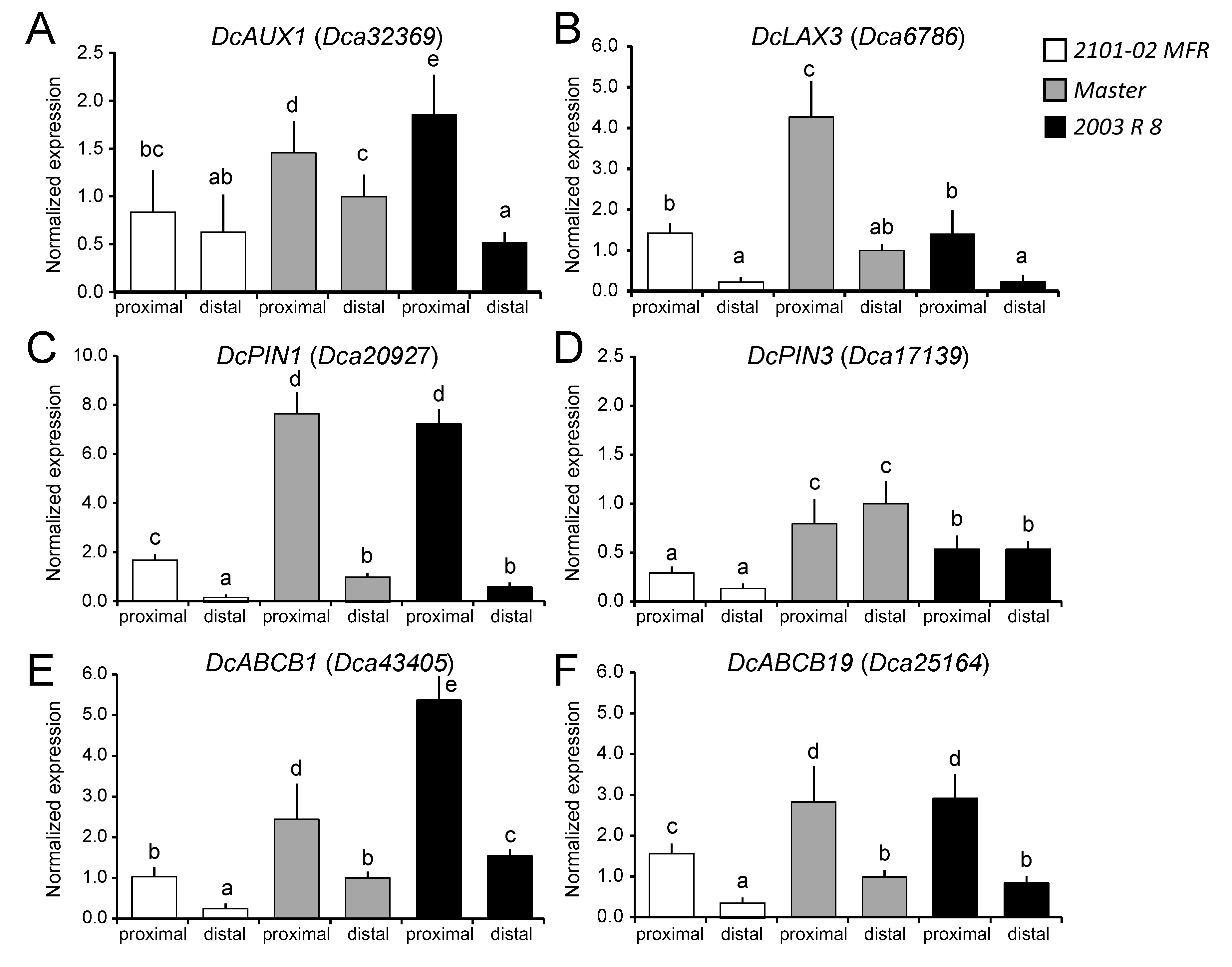

Supplement: FIGURE S3 — Real-time PCR quantification of the expression of selected transcripts related to auxin transport (auxin influx, A–B; auxin efflux, C–F) in mature leaves of carnation stem cuttings at harvesting time. Bars indicate normalized expression levels ± standard deviation relative to the distal region of the leaf in the ‘Master’ cultivar. Letters indicate significant differences between samples (P < 0.05). [file Image_3.TIF]

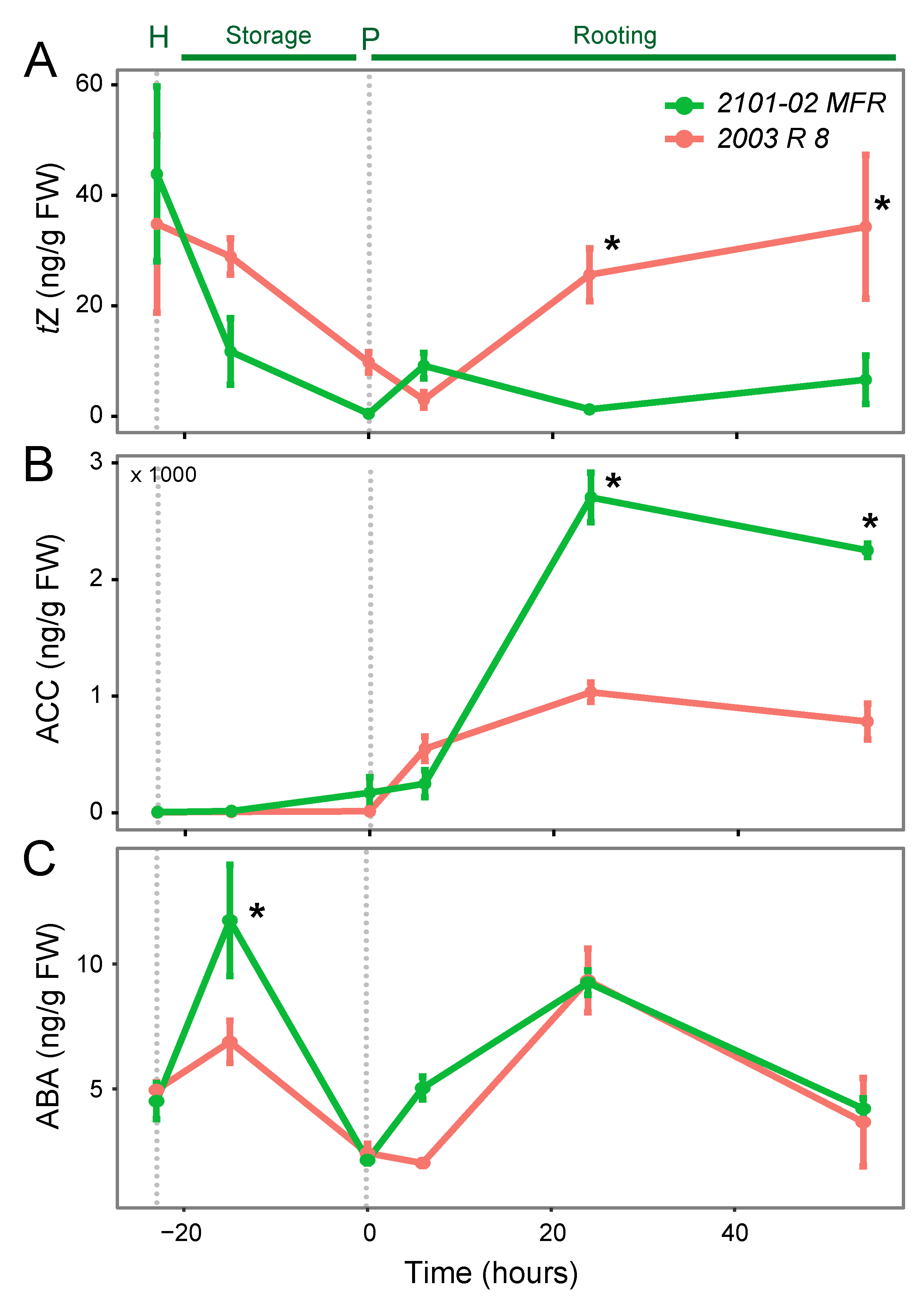

Supplement: FIGURE S4 — Endogenous levels of other key hormones in the stem cutting base during adventitious rooting. (A) trans-zeatin (tZ), (B) the ethylene precursor 1-aminocyclopropane-1-carboxylic acid (ACC), and (C) abscisic acid (ABA). Average ± standard deviation values are shown. Asterisks indicate significant differences (P < 0.05) over time for a given treatment. H, harvesting; P, planting. [file Image_4.TIF]

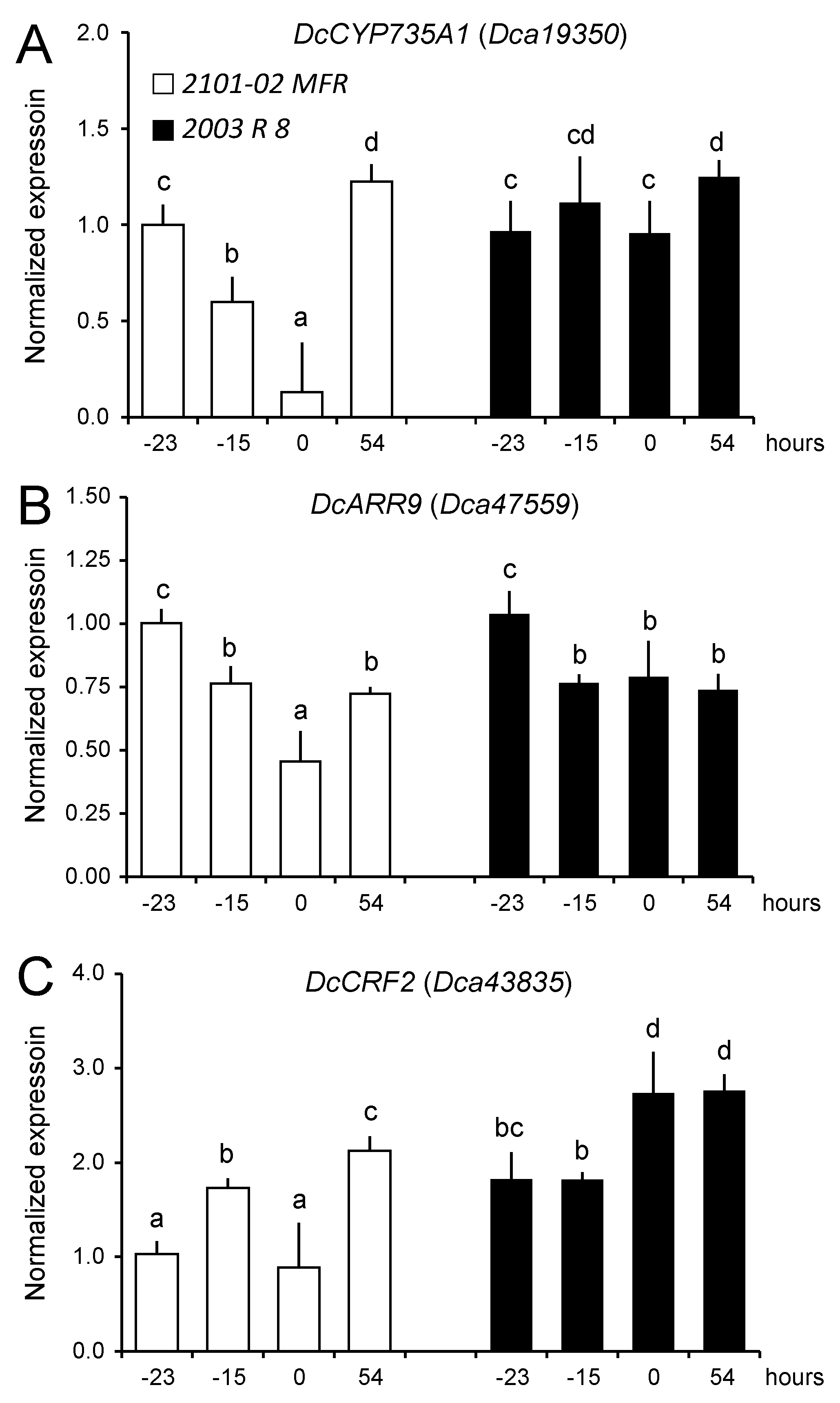

Supplement: FIGURE S5 — Real-time PCR quantification of the expression of selected transcripts related to CK biosynthesis (A) or signaling (B,C) in the stem cutting base during adventitious rooting. Bars indicate normalized expression levels ± standard deviation relative to the -23 h dataset in the “2101–02 MFR” cultivar. Letters indicate significant differences between samples (P < 0.05). [file Image_5.TIF]
