# Supplementary material for: Enhanced Conjugation of Auxin by GH3 Enzymes Leads to Poor Adventitious Rooting in Carnation Stem Cuttings
Source: Front Plant Sci. 2018 Apr 26;9:566. doi: 10.3389/fpls.2018.00566 (PMC5932754; doi:10.3389/fpls.2018.00566)
Supplement: TABLE S6 — Oligonucleotides used in this study. [file Table_1.DOCX]

**Table S1.- Oligonucleotides used in this study**

| **Gene** | **Accession number** | **Oligonucleotide sequences (5’→3’)** | | **Product (bp)** |
| --- | --- | --- | --- | --- |
| *DcABCB1* | *Dca43405* | TGCCGGAAAAACCATAGCACTTG | CTAATAGAAGTTGTCCTTCAGTGG | 107 |
| *DcABCB19* | *Dca25164* | GTTCATGAAGTTTCCAAGTATGC | CCAGCATGCAATTTCTGCGTAG | 87 |
| *DcARR9* | *Dca47559* | TTCATTCCAAGTTACAGCAGTTG | ATTCACCTCTACTTCCTGCTG | 130 |
| *DcAUX1* | *Dca32369* | GTATGGTCCCAATAATCCGTCC | CGTACCTCATTAGTGTATTGTAC | 119 |
| *DcCRF2* | *Dca43835* | AGAAGTCGGGTCTTATGTG | ACTAGACCAACCCGAATAC | 148 |
| *DcCYP735A1* | *Dca19350* | TCTCGCTTTTACGGGAAGAG | ACCATTAGCCATAAGTAAACCAC | 178 |
| *DcDAO1* | *Dca2789* | TGCCGTTCCATGGAGCAATGGA | TGTGCTTCAATGACCATTTCTGGC | 126 |
| *DcGH3.1* | *Dca37575* | TTCCGAGTTCCTTACTAGTTCG | GAAGGCTGTACAATAGTTGACG | 101 |
| *DcIAA19* | *Dca30890* | GGTTGTCTTAGCTTAGAAGATGC | CATGACTCAACAAACAGGTTCC | 128 |
| *DcLAX3* | *Dca6786* | ACAAGCAATAAGCTGGATAACAG | TCGAAACCATGTCATTCAGTGG | 127 |
| *DcPIN1* | *Dca20927* | CTAGTCTGTTTCAGGTGGAATTTG | GCAATGCCATGAATAAACCAAGAC | 118 |
| *DcPIN3* | *Dca17139* | CGGCGAATCTAAAGGTGCTAG | CCAAGTTTGTTCAGTCGAATTG | 114 |
| *DcTAR2a* | *Dca35926* | TGCTGGGATGCGCATTGGTTG | GAAACACCAATGCTACTCAGCT | 93 |
| *DcYUC1* | *Dca37589* | TCCGTAACTCGGTGCATGTAC | GTGAGATTCGCGATTAAGAGCA | 130 |
| *DcEF1α* | *Dca3524* | CTCCACCACTGGAGGTTTTGAA | CTTGACGATTTCTTCGTACCTC | 166 |
